# Forensic Brain-Reading and Mental Privacy in European Human Rights Law: Foundations and Challenges

**DOI:** 10.1007/s12152-020-09438-4

**Published:** 2020-06-20

**Authors:** Sjors Ligthart, Thomas Douglas, Christoph Bublitz, Tijs Kooijmans, Gerben Meynen

**Affiliations:** Department of Criminal Law, Tilburg University, Warandelaan 2, 5037AB Tilburg, Netherlands; Faculty of Philosophy, Oxford Uehiro Centre for Practical Ethics, University of Oxford, Oxford, UK; Faculty of Law, Universität Hamburg, Rothenbaumchaussee 33, 20148 Hamburg, Germany; Department of Criminal Law, Tilburg University, Warandelaan 2, 5037AB Tilburg, Netherlands; Willem Pompe Institute for Criminal Law and Criminology and UCALL, Utrecht University, Utrecht, Netherlands; Faculty of Humanities, VU University Amsterdam, De Boelelaan 1105, 1081HV Amsterdam, Netherlands

**Keywords:** Brain-reading, Mental privacy, Human rights, Criminal justice

## Abstract

A central question in the current neurolegal and neuroethical literature is how brain-reading technologies could contribute to criminal justice. Some of these technologies have already been deployed within different criminal justice systems in Europe, including Slovenia, Italy, England and Wales, and the Netherlands, typically to determine guilt, legal responsibility, or recidivism risk. In this regard, the question arises whether brain-reading could permissibly be used against the person's will. To provide adequate legal protection from such non-consensual brain-reading in the European legal context, ethicists have called for the recognition of a novel fundamental legal right to mental privacy. In this paper, we explore whether these ethical calls for recognising a novel legal right to mental privacy are necessary in the European context. We argue that a right to mental privacy could be derived from, or at least developed within in the jurisprudence of the European Court of Human Rights, and that introducing an additional fundamental right to protect against (forensic) brain-reading is not necessary. What is required, however, is a specification of the implications of existing rights for particular neurotechnologies and purposes.

## Introduction

A central question in the current neurolegal and neuroethical literature is how neuroscientific technologies could and should contribute to criminal justice. On the one hand, there is the use of neurotechnologies to *read* the subject’s brain in order to obtain information, such as brain-based diagnostics, lie and memory detection [1]. The results of some of such applications are already being used within different criminal justice systems in Europe, including Slovenia, Italy, England and Wales, and the Netherlands [2–5], typically to determine guilt or establish a neurological diagnosis relevant to legal responsibility or recidivism risk. On the other hand, there are applications aiming to *intervene* into persons’ minds/brains for various purposes, ranging from preventing crimes or enhancing eyewitness testimony to facilitating offender rehabilitation [6]. This paper addresses only the first type of application; it examines brain-reading, not brain-alteration.^1^

In criminal procedures, defendants and convicted offenders are not always willing to cooperate with the authorities. The question thus arises whether brain-reading could permissibly be used without valid consent in a forensic context. To date, forensic ‘brain-reading’ has typically been performed with the subject’s consent [7], but would it be factually possible, legally permissible and morally acceptable to deploy brain-reading *against the subject’s will* [8–10]? And if so, under what conditions? In the U.S. legal context, a right to ‘mental privacy’ that protects citizens from non-consensual *brain-reading* has been advocated [11]. In Europe, Marcello Ienca and Roberto Andorno have called for the recognition of a *novel* fundamental right to mental privacy [12], and so has Andrea Lavazza [13]. In this paper, we explore whether these *ethical* calls for recognising a novel *legal* right to mental privacy are decisive in the European context.

To answer this question, we explore the extent to which the European Convention on Human Rights (ECHR) protects against non-consensual brain-reading in matters of criminal procedure. Because the ECHR does not explicitly address brain-reading procedures – and neither do its *travaux préparatoires* – we need to establish the extent to which privacy interests regarding brain-reading are protected as part of more general current provisions. We argue that a right to mental privacy could be derived from, or at least developed within the jurisprudence of the European Court of Human Rights (ECtHR/the Court), most plausibly as part of the general right to ‘private life’ (Article 8 ECHR; reiterated in Articles 7 and 8 European Charter for Fundamental Rights and Freedoms). Although we acknowledge the importance and intricacies of *mental* privacy, we argue that introducing an *additional* fundamental right to protect against (forensic) brain-reading is not necessary. Moreover, introducing novel distinct rights may even be detrimental as it may lead to inconsistencies in privacy protection. Regulations of forensic investigations of brains should cohere with other regulations, in this case, regulations concerning other methods of criminal investigation, such as DNA-testing. However, in order to guarantee the effective enjoyment of mental privacy, a specification of the implications of the generic right to respect for private life for particular neurotechnologies and purposes is required. Drawing out such implications is a commonplace legal activity and does not require novel rights. Furthermore, our analysis shows that in its current understanding, some types of non-consensual brain-reading could be lawful – and this is an issue that may require political discussion as well as further development of the law.

The outline of this paper is as follows. To prepare the ground for our subsequent normative analysis, in section 2 we first briefly discuss current possibilities and limitations of brain-reading technologies in the criminal justice context. Subsequently, in section 3, we examine the ways in which the ECHR presently provides safeguards against forensic brain-reading through the rights to freedom of thought and privacy, as well as the right not to incriminate oneself. Based on our findings, in section 4, we assess, from both a legal and ethical perspective, two recent calls for creating a novel fundamental right to mental privacy.

## Brain-Reading in Criminal Justice: What Is Possible?

Brain-reading promises to yield information relevant for the law, especially criminal law. Recent research focuses on neuroimaging technologies, such as magnetic resonance imaging (MRI), functional magnetic resonance imaging (fMRI), computed tomography (CT) and electroencephalography (EEG). Two types of neuroimaging can be distinguished: structural and functional [14]. While structural neuroimaging, such as MRI and CT, reveals the biological structure of the brain (i.e. brain anatomy), functional neuroimaging, such as fMRI and EEG, measures brain activity (i.e. brain physiology). Progress in these technologies, and the computations powering them, has revolutionised the way we understand the human brain. Moreover, to some extent, imaging results also allow inferences to be drawn regarding a person’s mental states. Thus, neuroimaging reads not only brains, but sometimes also minds. In particular, neuroimaging can detect mental reactions of persons to visual and other stimuli to which they are exposed, and some of these reactions are automatic, non-conscious, not under the willful control of the person, or a combination thereof. This may be an attractive feature of neuroimaging for some forensic purposes, for example, as a way of bypassing deliberate attempts to deceive the authorities.

A range of potentially relevant applications are currently under development. Among the most promising for forensic purposes are: (1) brain-based lie detection [15]; (2) detection of whether a stimulus is novel or familiar to the person (‘concealed information test’) [16]; (3) assessment of mental capacities and performance, as well as mental disorders, e.g. for the determination of fitness to stand trial, assessment of culpability, the applicability of the insanity defence, or for substantiating claims of victims [17]; and (4) identification of preferences, likes or dislikes, or other character traits or dispositions, from aggressiveness to paedophilia [18, 19].

Some of these applications are already in use in Europe. For example, as the studies of Hafner, Catley and Claydon, and De Kogel and Westgeest show, neuroimaging has been used in criminal cases in the context of, inter alia, establishing legal insanity, i.e. for forensic psychiatric assessments of defendants [2, 4, 5]. To be clear, many of these applications are still in laboratory stages and not (yet) at a stage that allows their admission in legal proceedings.^2^ Nevertheless, some show promising (preliminary) results. For instance, a study by Aharoni and colleagues found a “neurocognitive biomarker for persistent antisocial behaviour”, which they extended to “neuroprediction of future arrest” [20]. The researchers tested ACC activity in subjects (*n* = 96) through an impulse control task while their brain was scanned via fMRI. They then related the brain data and other risk factors to the rearrest rate four years after release. They found that ACC activity predicts rearrest. The researchers note that these measures will likely not outperform other measures, but still they may add some predictive value and so have a role to play in a larger assessment battery. An interesting example of such an ‘interactive approach’, is the study of Delfin et al., who performed a long-term follow-up study on the prediction of recidivism of forensic psychiatric patients, using both traditional risk assessment tools and resting-state SPECT data. One of the “incremental effects of neuroimaging data” they reported, was the increase of accuracy rate from 64% to 82% [21].

These and related matters could become relevant during criminal (but also other) proceedings at some point. Brain data will likely be used in combination with other data, for example, to inform psychiatric evaluations. Given that epistemic access to other minds is in principle limited, and since current risk assessment tools are far from perfect [22], neuroimaging may make a significant contribution [19, 21, 23], especially with respect to people who refuse to cooperate, malinger, or simulate – or are suspected of doing so.

Two severe limitations should also be noted. First, the neurobiological studies provide findings at group level. Applying them to individuals requires further information and inferences. Second, as yet, the risk of participants actively sabotaging certain measurements, e.g., by moving their heads in the brain scanner or employing other countermeasures, cannot be entirely excluded [24, 25].

Despite important limitations of current technologies for brain-reading, we should look beyond today’s horizon, anticipating developments and considering potential legal and ethical implications of non-consensual forensic brain-reading for the subject’s privacy interests. In the U.S., a debate about these issues has been ongoing for over a decade [26, 27], and we feel it is time to transfer it to the European legal context. The following section explores whether and, if so, to what extent the ECHR protects against non-consensual neuroimaging in criminal justice.

## Exploring Current Legal Protection: The European Convention on Human Rights

### Introduction

In this section we analyse the extent to which the European Convention on Human Rights (ECHR) protects against non-consensual neuroimaging in criminal justice. We focus on those rights that may be engaged by compulsory governmental acquisition of (personal) information. These are the right to freedom of thought, conscience and religion (Article 9 ECHR), the right to respect for private life (Article 8 ECHR), and the right not to incriminate oneself (Article 6 ECHR).

### The Right to Freedom of Thought (Article 9 ECHR)

We begin with the strongest, because absolute, right: the right to freedom of thought, pursuant to Article 9(1) ECHR. This right comprises two dimensions, internal (the *forum internum*) and external (the *forum externum*). Whereas the internal dimension is absolute and may thus not be restricted, infringements of the external dimension could, under certain circumstances, be justified (Article 9(2) ECHR). Note, that the *forum externum* only comprises the manifestation of religion and (religious and non-religious) beliefs, not of thoughts. Yet, as Malcom Evans notes, thoughts can be ‘mani-fested’ through actions, primarily via speech and expression, covered by Article 10 ECHR [28, p. 285]. Since forensic brain-reading will normally not (intend to) reveal the individual’s religion or beliefs, we will focus here on the freedom of *thought* as guaranteed by the *forum internum* of Article 9 ECHR.

According to the *Human Rights Handbook* of the Council of Europe, the internal dimension of Article 9 ECHR seeks at its most basic level “to prevent state indoctrination of individuals by permitting the holding, development, and refinement and ultimately change of personal thought, conscience and religion” [29, p. 18].^3^ Accordingly, the *forum internum* of the right to freedom of thought seems particularly relevant for neurotechnologies that *intervene* in the subject’s mind or brain, such as neuroenhancement through (non-)invasive brain stimulation [9, 32, 33]. In addition, according to the preparatory work on Article 9 ECHR, the right to freedom of thought, conscience and religion also intends to protect “not only from ‘confessions’ imposed for reasons of State, but also from those abominable methods of police enquiry or judicial process which rob the suspect or accused person of control of his intellectual faculties and of his conscience.”^4^ Vermeulen and Roosmalen state that freedom of thought prohibits any form of compulsion to express thoughts [31, p. 738–739]. Similarly, Harris et al. write that States may not require disclosure of someone’s personal convictions [30, p. 573–5]. Since brain-reading might be thought to involve, or to be equivalent to, the expression or disclosure of thoughts, some forms of brain-*reading* might potentially raise issues under the right to freedom of thought as well [34, 35].

Whether brain-reading indeed infringes the right to freedom of thought depends on whether the targets of these applications could be classified as ‘thoughts’ within the meaning of Article 9 ECHR. The definition of ‘thought’ in this respect is, however, unclear [32]. Case-law indicates a narrow approach to the *forum internum* [29, 30]. For example, according to the Grand Chamber of the ECtHR, “The right to freedom of thought, conscience and religion denotes only those views that attain a certain level of cogency, seriousness, cohesion and importance.”^5^ Yet, at the same time, a broader interpretation has been advocated, covering a wider range of mental states including emotions, dreams, and more trivial thoughts such as which car to buy, or which movie to watch [34–36].

Since the ECtHR has not set out to define ‘thoughts’, and the same is true for the other international human rights courts, the precise scope of the right remains unclear. Historically, ‘thought’ has been introduced to the UN Declaration alongside the older ‘religion and conscience’ to broaden the scope and protect ‘free thinking’ in its entirety, including (modern) scientific, philosophical, and political thoughts.^6^ This speaks for a broader understanding of ‘thought’. However, a de facto impossibility of altering and revealing thoughts in a direct way has always been a background assumption of the law [32, 34, 36].^7^ Neither drafters, nor judges, nor commentators have seriously anticipated the possibilities that neuroscience may offer in this respect.^8^ Historical statements or parallels are thus not by definition a convincing guide for a contemporary analysis of the right.

In any case, if a particular brain-reading application reveals information about, for example, the subject’s political preferences, scientific thoughts, or philosophical ideas that are protected under Article 9 ECHR, this fundamental right will offer legal protection against non-consensual use. However, deploying present neuroimaging applications, at least in the context of forensic diagnostics and risk assessment, typically does not disclose the defendant’s or convicted offender’s convictions or beliefs about morality, politics, or religion. The as-yet-undecided question is whether reading out *other* kinds of thoughts – from ordinary preferences to mundane beliefs – are, or ought to be protected as well [an argument to this end is developed in 34–36]. Moreover, whether lie or memory detection qualifies as *thought* detection is even less clear.

In such applications, neuroimaging indicates whether or not a person performs a particular kind of mental task, i.e. if she is remembering, calculating, daydreaming or deceiving. It discloses the process of thinking, but does not necessarily reveal the *content* of ‘thoughts’. If detecting types of mental acts qualifies as detecting types of thought, *forum internum* protection may arise. However, whether memories, lies and emotions are covered by the *forum internum* of Article 9 ECHR as ‘thoughts’, is not clear at the moment. These are presently open questions that deserve further research. In this regard, in our view, the following issues could be relevant.

First, for the evaluation of whether certain forensic brain-reading applications, such as memory and lie detection, would (or should) fall within the scope of freedom of thought, an analogy with obligatory witness testimony might be helpful. The use of such testimonies in criminal law is broadly accepted, also by the ECHR,^9^ as normally not raising any issues under the freedom of thought, but, potentially, under another right: the right (not) to express oneself pursuant to Article 10 ECHR.^10^ In ordinary questioning, the law imposes a *behavioural duty* on witnesses to testify truthfully, and this requires them to perform a mental task (remembering) and reporting the results. A neuroimaging method (‘memory detection’) could consist in showing the person e.g. a picture. The witness’ behavioural duty might then be to look at it. If imposing a duty on witnesses to testify truthfully is permissible under Article 9 ECHR, why should coercive brain-reading, e.g., memory detection, be prohibited? One could argue though, that memory detection requires the subject to attentively observe the presented stimuli, and react to them at a given time – forcing him to remember, which might infringe on free thinking more severely than a regular witness examination does. However, whether the Court would be inclined to follow such a line of reasoning remains to be seen.^11^

Secondly, although a particular memory or lie detection test focuses on specific questions (e.g., do you recognise the defendant?), much (additional) brain data could be acquired through the process. For example, an fMRI of a person’s brain in the context of lie detection may yield a great deal of information regarding the subject’s brain activity: information connected, but also not connected with the particular questions. Although at present it is unlikely that this information would include information about the content of a person’s thoughts or beliefs, future re-analysis of the acquired data might yield such information. By contrast, witness statements are not normally open to such reinterpretation.

Altogether, if brain-reading reveals thoughts, the right to freedom of thought will likely offer (strong) legal protection. But what qualifies as a thought for the purpose of Article 9 ECHR remains unclear. On narrow understandings, it is doubtful whether existing forms of brain-reading indeed reveal thoughts or beliefs. On broad understandings, it is plausible that they do.

### The Right to Privacy (Article 8 ECHR)

Meanwhile, every mental state or process that does not fall under Article 9 ECHR may be protected by Article 8 ECHR. Article 8(1) ECHR protects the right to respect for one’s private life. The ECtHR acknowledges that everyone has the right to live privately, away from unwanted attention. According to the Grand Chamber, “it would be too restrictive to limit the notion of “private life” to an “inner circle” in which the individual may live his or her own personal life as he or she chooses, thus excluding entirely the outside world (…). Article 8 thus guarantees a right to “private life” in a broader sense, such that it includes, for example, a right to lead a “private social life”, that is, the possibility for the individual to develop in private his or her social identity.”^12^ The notion of private life does, however, not lend itself to exhaustive definition.^13^ Whether particular data recorded and retained by the government is protected, depends on “the specific context in which the information at issue has been recorded and retained, the nature of the records, the way in which these records are used and processed and the results that may be obtained.”^14^ It is beyond dispute that it includes protection from collection, storage and disclosure of personal data^15^ – as becomes manifest in Article 8 of the European Charter for Fundamental Rights and Freedoms as well. In this regard, ‘personal data’ is defined as information regarding an individual who could be identified on the basis of the data and other information in the public domain.

Since at present no case-law of the ECtHR exists on the use of non-consensual forensic neuroimaging in light of Article 8 ECHR, it may be helpful – in order to explore the legal protection afforded to forensic brain-reading under this provision – to compare forensic neuroimaging with other methods of criminal investigation about which case-law does already exist [37]. Consider case-law on forensic fingerprinting and DNA-testing, which may provide some helpful insights. These methods are analogous to forensic brain-reading in the respect that the data they produce relates to biological features of the individual (e.g. the structure of one’s fingertip or brain, DNA and hemodynamics in blood and brain electricity). Furthermore, according to the ECtHR, DNA and fingerprints contain *unique* information which relates to an identified or identifiable individual, therefore containing protected personal data.^16^ The same is most probably true for neuroimaging since nobody’s brain anatomy is identical to anyone else’s, even in the case of monozygotic twins [38]. Furthermore, brain activity (more specifically, functional brain connectivity) appears also to be unique for any individual, “similarly to a fingerprint” [39]. Fingerprints, DNA and neuroimaging data are in this respect similar. Therefore, in considering non-consensual forensic brain-reading in light of Article 8 ECHR, we take into account case-law of the ECtHR on forensic fingerprinting and DNA-testing. However, we also wish to point to two important differences. First, DNA-testing and fingerprinting are used for purposes of *identification*, whereas forensic brain-reading is not. Second, DNA-testing and fingerprinting do not disclose mental states, whereas forensic brain-reading does. Here, analogies end.

In light of the Court’s case-law on yielding personal data through DNA-testing and fingerprinting, it is clear that obtaining, retaining and using protected personal neurodata without the subject’s consent *infringes* the right to respect for private life [37]. The crucial question is whether this infringement can be *justified*. According to Article 8(2) ECHR, such infringement could be justified if it (1) is based on a foreseeable and accessible legal ground, (2) serves a legitimate interest and (3) is necessary for and proportionate to the aim (i.e., legitimate interest) being pursued.

Regarding forensic brain-reading, the first two requirements need not be an obstacle as long as its use is regulated by sound legislation, based on which it can be applied in the legitimate interest of national security, the detection and prevention of crime or the protection of the rights and freedoms of others (e.g., by contributing to the assessment of guilt, criminal responsibility or a risk of recidivism). Perhaps, member states need to pass new laws, setting out criteria for the use of such data. But this is not an obstacle in-principle. However, whether non-consensual forensic neuroimaging will also be necessary and proportionate with the interest pursued (third requirement), is more open to debate.

Ultimately, whether an infringement of the right to respect for private life is necessary and proportionate, depends on the seriousness of the infringement. The more serious the infringement, the more important its aims should be. In the context of recording, retaining and using personal data, the seriousness of the infringement largely depends on the amount and privacy-sensitivity of the data concerned.^17^ For instance, the ECtHR considers cellular samples to be ‘highly personal’, containing much sensitive information about (the health of) an individual and a unique genetic code of great relevance to both the individual and his relatives.^18^ DNA-profiles contain a more limited amount of personal data, but nonetheless contain ‘substantial amounts of unique personal data’, which enables identification of genetic origins and relationships between individuals.^19^ In addition, the Court remarks that – bearing in mind the rapid pace of developments in genetics and information technology – it cannot discount the possibility that future private-life interests may be adversely affected in novel, yet unforeseeable ways.^20^ Whereas fingerprints contain not as much personal information as cellular samples and DNA profiles, they nevertheless constitute personal data containing identifying features.^21^

Similarly, one might argue that the results of different neuroimaging applications will also differ in their levels of privacy-sensitivity. For example, identifying the mere recognition of a particular car, gun or person through memory detection, seems generally to involve less sensitive information than the diagnosis of (terminal) brain cancer or a high risk of criminal behaviour. Some future neuroimaging technologies might even yield more sensitive data, about specific moral or political commitments, feelings about personal relationships, or fundamental personality traits and proclivities (in some cases this could also raise an issue under the right to freedom of thought, discussed above). In contrast to these possible future neuroimaging technologies, however, the data yielded by current and near-future applications on which we focus here, like diagnostics, neuroprediction and memory and lie detection, will likely be somewhat more mundane. In the context of criminal justice, they may include, for example, information regarding whether the defendant recognises a particular gun or lies about a specific alibi at a specific time. Of course, they may also be more sensitive, such as whether someone suffers from a specific psychiatric or neurological disorder.

Whether data of this kind would, in general, be significantly more sensitive than the information cellular samples contain, can be debated [37, 40, 41]. After all, cellular samples contain a wide range of sensitive personal data, e.g., regarding health, ethnic origin and genetic relationships. Most of this information will not be used in the context of a criminal case: only a specific element that enables biometric identification will be extracted and analysed. Nevertheless, for the ECtHR it is significant that, although only a small part of the data will be used, all other sensitive information is obtained and retained as well when a cellular sample is taken:

“In addition to the highly personal nature of cellular samples, the Court notes that they contain much sensitive information about an individual, including information about his or her health. Moreover, samples contain a unique genetic code of great relevance to both the individual and his relatives. (…) Given the nature and the amount of personal information contained in cellular samples, their retention *per se* must be regarded as interfering with the right to respect for the private lives of the individuals concerned. That only a limited part of this information is actually extracted or used by the authorities through DNA profiling and that no immediate detriment is caused in a particular case does not change this conclusion.”^22^

The extent to which present forms of brain-reading involve such a ‘trawl-like’ collection of data will depend on the particular test and its technology. For example, the information yielded through a concealed information test, applied with EEG, can be quite restricted by the specific purpose of the test, e.g.: do you recognise this particular knife? Electrodes on the subject’s scull measure particular brain electricity and the analysis focusses on a specific brain wave (P300), indicating whether or not one recognised the presented stimuli [42]. If the same test is applied with fMRI, however, the acquired information will probably be less restricted, since the fMRI-scanner does not measure specific brain activity, but makes a functional image of the *whole* brain in action. Depending on the analytical methods that are used to interpret the data, a prima facie limited fMRI-memory-detection-test might actually provide a great deal of other information, e.g., regarding medical pre-dispositions, risk of future offending, or even sexual proclivities. In addition, if new technologies and decoding systems will enable unfiltered, trawl-like detection of particular thoughts or emotions, the acquired data will no longer be restricted by the purpose of the test, but by those things the defendant feels or thinks about [37, 43].

In conclusion, it is important to note that despite the highly sensitive nature of cellular samples, the ECtHR has repeatedly ruled that the compulsorily taking, examination and retention of cellular material and DNA-profiles for identification purposes, was – with a certain discretion for the State – necessary and proportionate for the detection and prevention of crime and the protection of the rights and freedoms of others.^23^ In doing so, the Court tailored the generic right to respect for private life to the intricacies of forensic DNA-testing. Similarly, we argue, a legal approach tailored to forensic brain-reading can be developed under Article 8 ECHR as well. Although DNA-testing and brain-reading are in several respects disanalogous, case-law on DNA may provide helpful insights for how to develop such an approach, but cannot be applied to forensic brain-reading directly. Nonetheless, it provides a rough direction. At least, we can safely conclude that non-consensual (forensic) brain-reading is covered by the general fundamental right to privacy. How the law should treat specific infringements within the context of Article 8 ECHR, is, however, an open question.

### The Right Not to Incriminate oneself (Article 6 ECHR)

Finally, let us turn to the right against self-incrimination—a right that normally only applies to defendants, and not, for example, to witnesses, or convicted offenders. According to the Grand Chamber of the ECtHR, the right to remain silent and not to incriminate oneself are generally recognised international standards which lie at the heart of the notion of a fair trial contained in Article 6 ECHR.^24^ Their rationale lies, inter alia, in the protection of defendants against improper compulsion by the authorities, thereby contributing to the avoidance of miscarriages of justice and to the fulfilment of the aims of the right to a fair trial as laid down in Article 6 ECHR.^25^

According to the Grand Chamber, “the right not to incriminate oneself is primarily concerned with respecting the will of an accused person to remain silent and presupposes that the prosecution in a criminal case seek to prove their case without resorting to evidence obtained through methods of coercion or oppression in defiance of the will of the accused.”^26^ Accordingly, respecting the *will* of a defendant not to produce any self-incriminating evidence is the primary aim of this right [44, p.266]. It does not protect against the eliciting of an incriminating statement per se, but against the obtaining of evidence *by means of coercion or oppression.* ^27^ Ultimately, evidence which has been obtained without respecting the will of the accused not to produce the evidence, may not be used against him in his criminal procedure [45, 46].

In the *Saunders* landmark case, the Court formulated an important limitation of the scope of the right against self-incrimination:

“it does not extend to the use in criminal proceedings of material which may be obtained from the accused through the use of compulsory powers but which has an existence independent of the will of the suspect such as, inter alia, documents acquired pursuant to a warrant, breath, blood and urine samples and bodily tissue for the purpose of DNA testing.”^28^

Case-law on the precise scope of this exception is, however, not clear. Whereas the Court approaches ‘documents acquired pursuant to a warrant’ as material which has an existence independent of the will of the suspect, therefore falling beyond the scope of the right not to incriminate oneself, the obligation to provide certain unspecified documents, which could only be obtained with the cooperation of the person concerned, did violate this right.^29^ Therefore, what the Court precisely means with material *which has an existence independent of the will of the suspect*, is debatable. One interpretation is that information which can be acquired without the cooperation of the suspect, so which can be obtained independently of the defendant’s will, exceeds the scope of the right [44, 45]. In this view, the question is whether the defendant’s *cooperation is necessary* in order to obtain the information. If so, the information and the coercive acquisition of it are covered by the right against self-incrimination.

Such a ‘means-based’ approach, in which the *manner of obtaining* evidence is decisive rather than the *nature* of the acquired information, makes sense in light of the primary aim of the right: respecting the *will* of an accused person not to *provide* self-incriminating evidence [45]. If the evidence can only be acquired with cooperation of the suspect who refuses to cooperate, the evidence can only be obtained through breaking the suspect’s will not to provide the information (e.g., by making severe threats). Such a way of obtaining evidence is, however, not allowed under the right not to incriminate oneself.

On this view, whether the right against self-incrimination protects from non-consensual forensic brain-reading depends on whether the brain-reading application requires the subject’s cooperation. In general, functional neuroimaging during which the subject must perform a certain task does indeed require cooperation. In the context of memory and lie detection, for instance, the subject must attentively observe certain stimuli or press a yes-or-no-button after each question.^30^ Furthermore, the subject must refrain from manipulating the test using countermeasures. Hence, the results of such functional neuroimaging applications could not be obtained from any other source than the cooperating subject himself, and are thus, in general, protected from non-consensual acquisition.

By contrast, in principle, structural imaging technologies do not require cooperation of the subject. At least in theory, the intended results can be obtained while the subject is under general anaesthesia. Therefore, non-consensual structural neuroimaging, for example in the context of diagnostics or neuroprediction, does not, according to this view, fall within the scope of the right against self-incrimination.^31^ As a consequence, the right not to incriminate oneself seems mainly to protect against non-consensual *functional* neuroimaging that requires cooperation, such as the detection of lies and memories.

Notably, however, the right against self-incrimination only applies to an individual who is ‘charged with a criminal offence’.^32^ Accordingly, it will normally not apply to *witnesses* in a criminal case.^33^ Secondly, the rights and principles of Article 6 ECHR do not apply to procedures regarding preventive measures *without any concrete suspicion*,^34^ such as the use of neuroprediction or brain-based diagnostics in the context of involuntary admission in health care. Non-consensual neuroimaging in these contexts will thus not be covered by the right not to incriminate oneself. Finally, since procedures regarding the *execution* of criminal sanctions do not involve the determination of a criminal charge, Article 6 ECHR does not apply in such cases.^35^ As a consequence, the right against self-incrimination will not cover non-consensual brain-reading in the context of the execution of criminal sanctions, such as brain-based risk assessment of prisoners who request parole, or the use of lie detection to verify whether a convicted offender complies with his conditions of probation.

### Synthesis

In this section we explored the extent to which the ECHR protects against the non-consensual acquisition of (personal) information through forensic brain-reading. The right to freedom of thought (Article 9 ECHR) will only offer protection if neuroimaging reveals ‘thoughts’ or ‘beliefs’. Whereas brain-reading for the purposes of forensic diagnostics and risk assessment typically does not reveal thoughts, futuristic real-time thought reading could elicit protection under Article 9 ECHR. Whether present forms of memory and lie detection yield any thoughts, and are thus protected by Article 9 ECHR, is, as yet, less clear. It is clear though, that reading a person’s brain (activity) with neuroimaging is covered by the generic right to privacy (Article 8 ECHR). While case-law on DNA-testing might suggest that some forms of non-consensual brain-reading could be lawful in this context, the (case-)law should develop its own approach for this type of data-acquisition, within the doctrine of Article 8 ECHR. Finally, we explored the implications of the right against self-incrimination (Article 6 ECHR), and concluded that this right, in general, mainly protects against deploying brain-reading (in the determination of a criminal charge) if the test at issue requires cooperation of the subject. The right will normally not cover structural brain-reading applications (such as MRI and CT for the purposes of diagnostics or risk assessment) and nor will it cover the use of functional brain-reading tests like lie and memory detection, when deployed in the *execution* of criminal sanctions.

## Discussion: The Desirability of a Novel Fundamental Right to Mental Privacy

As discussed above, the current framework of European human rights does not universally prohibit the use of non-consensual brain-reading in criminal justice: particular brain-reading applications could be lawful. Let us now look briefly at the most prominent arguments for explicitly recognising a fundamental right to mental privacy that would provide fuller protection against non-consensual forensic brain-reading.

### The Special Features of Brain Data

In a recent paper, Marcello Ienca and Roberto Andorno discuss the implications of emerging neurotechnologies in the context of European human rights, and suggest that existing human rights may not be sufficient to respond to these emerging issues. As to the collection of brain data and the right to mental privacy, they argue that.

“the special nature of brain data, which relates very directly to one’s inner life and personhood, and the distinct way in which such data are obtained, suggest that specific safeguards will be probably needed in this domain” [12, p. 14].

They further argue that.

“current privacy and data protection rights are insufficient to cope with the emerging neurotechnological scenarios. Consequently, we suggest the formal recognition of a right to mental privacy” [p. 15].

One of the special features of brain data, they maintain, is that the information to be protected is not easily distinguishable from the source that produced it, i.e. the subject’s neural processing. Because of this “inception problem”, wider privacy and data protection rights are needed, which not only protect the recorded and retained *information*, but also the *source* of that information since they may be inseparable. In order to implement this, the authors argue, “we would need wider privacy and data protection rights that can be also applied at a higher and chronologically antecedent level: *our neural activity*.”

In response, we note that the existence of an “inception problem” can be questioned. We might distinguish between *information* about neural/mental states, and the neural/mental states themselves (the *source* of that information).^36^ It is not clear that the relationship between source and information here is any different to the relationship that exists with respect to other forms of data.

Moreover, the authors do not explain *why* such an inseparable relation between source and information would justify extraordinary privacy protection, or why the existing right to privacy is inadequate in this respect. Even if they have correctly identified a descriptive feature of information acquired through brain-reading – that it cannot easily be distinguished from its source – there is a further question as to whether this descriptive feature ought to be given legal significance. Arguing that it ought, is unlikely to be straightforward given that genetic information presumably also raises the “inception problem”, which information is as yet approached under the generic right to privacy pursuant to Article 8 ECHR.

To avoid these concerns, Ienca and Andorno could shift the focus away from the inception problem, and towards their claim that brain data “relates very directly to one’s inner life and personhood”. However, while this descriptive claim seems plausible, the supposed normative implications seem again less self-evident than the authors suggest. It is not clear why the results of brain-reading per se relate more to one’s inner life and personhood than current (non-consensual) methods of gathering information in the context of criminal justice, such as DNA-testing, psychiatric diagnostics and risk assessment, which regularly concern highly sensitive and even intimate issues, from ethnic origins over biographical and psychological development of persons to disorders or sexual dysfunction. For example, should we consider ‘the recognition of a particular gun’, identified through EEG-based memory detection, to be more personal than one’s ethnic origin disclosed through DNA-testing, just because the former concerns ‘brain data’?

Note, finally, that if (the interpretation of) brain-reading results will enable the future acquisition of information about particular (sensitive) thoughts, the use of non-consensual brain-reading may trigger stronger legal protection by the existing right to freedom of thought (Article 9 ECHR). Up to that point, the current right to privacy seems able to cover all privacy-interests of those who are subjected to forensic brain-reading.

### Autonomy

Another argument that advocates special privacy protection for brain-reading, is the argument from autonomy. In discussing the implications of detecting mental states through neural prosthetics, Andrea Lavazza underlines the (ethical) premise that the absolute privacy of one’s mental states and content, is one of the most valuable and inviolable human rights. This is due to its close link to autonomy:

“privacy, understood as the secrecy of one’s brain data and mental contents, is key to a free conduct, because autonomy is exercised not only in public but also in private. Being spied on through mind-reading reduces the subject’s autonomy in the Kantian sense, that is, the subject can be limited in self-imposing her own norm of conduct, free of external pressures and conditioning (which are only avoided by keeping one’s thoughts private)” [13, p. 4].

From the perspective of criminal law, however, this argument could be debated. First, the mere neurotechnological acquisition of particular personal data within the context of a criminal procedure, such as whether one suffers from traumatic brain injury or recognises a particular gun, would probably not restrict the subject in ‘the right to make choices as to how to lead one’s own life’, which is how the ECtHR defines the right to personal autonomy.^37^ Moreover, restricting autonomy is not uncommon in criminal law, e.g., by imposing the duty on a witness to testify truthfully. Investigating whether this duty is observed by a witness is then hardly a further relevant setback to autonomy. So, while autonomy is certainly a background consideration, and an important principle underlying the interpretation of Article 8 ECHR,^38^ it appears too broad to generate more concrete demands on privacy protection in this particular and exceptional scenario. Secondly, since current ‘mind reading’ neuroimaging requires co-operation on the part of the subject, the subject in fact always keeps (some) control over whether or not the authorities acquire the information they seek to obtain [50]. But even if we assume that coercive brain-reading could completely circumvent a person’s will not to reveal particular, undisclosed information, the question arises why non-consensual disclosure of such previously undisclosed information through neuroimaging justifies stronger legal protection than the disclosure of information through other means, such as tapping telephones and reading diaries. Again: privacy is an important good, but why is mental privacy qualitatively different?

In our view, existing (European human rights) law already rules out the obviously impermissible use of brain-reading technologies, and provides the resources for deciding on ‘grey area’ technologies. Hence, although the explicit recognition of a novel right to mental privacy may underline the importance of this particular human interest, it is at best superfluous to provide adequate legal protection. What is needed for this purpose, however, is a legal construal of the existent rights tailored to the use of non-consensual brain-reading that fits within the present doctrines, such as Article 8(2) ECHR. Such tailoring is, however, a commonplace legal activity. It is necessary to determine the strength of privacy protection with respect to various brain-reading techniques, on a more fine-grained level. Legal systems can develop this with respect to particular purposes and methods, and this process of shaping the right to privacy is to some extent open to political debate and ethical oversight. But it will, ultimately, be a balancing between legitimate interests on either side.^39^ A novel right to mental privacy would not change this, it would encounter the same conflicting interests.

## Conclusion

Neuroimaging technologies enable the reading out of different types of information from our brains, offering a new, potentially valuable tool for non-consensual data-acquisition in the context of criminal procedure (in the future). Although some forms of non-consensual forensic brain-reading could be lawful under the current framework of European human rights, existing fundamental rights do rule out the obviously impermissible uses of brain-reading, and provide the resources for deciding on others. In this paper, we focussed on Articles 6, 8 and 9 ECHR. In addition, as briefly mentioned, the prohibition on ill-treatment pursuant to Article 3 ECHR shall also set some fundamental boundaries to the use of forensic brain-reading. Altogether, in order to effectively guarantee the right to mental privacy, a novel fundamental right is, in our view, not necessary. Instead, we argue, developing a legal approach that tailors the existing doctrine of fundamental (privacy) rights to the use of non-consensual brain-reading, would be desirable and deserves closer examination.

## Footnotes

1 Whereas ‘reading’ brains typically raises questions as to mental *privacy*, brain-alteration does so regarding mental *integrity*. Although it may be possible to subsume mental privacy and mental integrity within a single over-arching concept—perhaps mental autonomy—we do not wish to take a stance on this here.

2 Here, we wish to note a European difference to the USA dominated discourse. The *Frye* or *Daubert* standards, according to which new technologies must have “consensus” among experts in the field, are not shared by all European, especially civil law jurisdictions. For instance, in Germany, it suffices that a piece of evidence potentially helps to find the truth.

3 Cf. [30, p. 537] and [31].

4 European Commission of Human Rights, *Preparatory work on Article 9 of the European Convention on human rights*, Strasbourg, 16th August 1959, p. 3–4. Cf. Human Rights Committee, General comment no. 22, 30.07.1993: “No one can be compelled to reveal his thoughts or adherence to a religion or belief.”

5 ECtHR (GC) S.A.S. v France, appl.nos. 43,835/11 (1 July 2014), para 55; ECtHR (GC) izzettin Dogan and others v. Turkey, appl.nos. 62,649/10 (26 April 2016), para 68.

6 W.A. Schabes, *The Universal Declaration of Human Rights. The travaux préparatoires*, New York: Cambridge University Press 2013, pp. 1766, 2489, 2500 (Soviet Union), 1766–1767 (France) and 2499 (Uruguay).

7
*Id*., pp. 1766, 1768 (France) and 2518 (Argentina).

8 E.g. Vermeulen & Roosmalen 2018, p.738: “It is true that thoughts (…) – as long as they have not been expressed – are intangible and only realise their full potential by expression in *foro externo*.”

9 E.g ECtHR Wanner v. Germany, appl.no. 26892/12 (23 October 2018).

10 The obligation to take a religious oath, however, could violate article 9 ECHR: ECtHR Buscarini and Others v. San Marino, appl.no. 24645/ 94 (18 February 1999).

11 See S. Ligthart, Freedom of Thought in Europe: Do advances in brain-reading technology call forrevision?, J Law Biosci (accepted for publication).

12 ECtHR (GC) Bărbulescu v. Romania, appl.no. 61496/08, § 70.

13 ECtHR (GC) Paradiso and Campanelli v. Italy, appl.no. 25358/12, § 159 (24 January 2017).

14 ECtHR (GC) S. & Marper v. UK, appl.nos. 30,562/04, 30,566/04, § 66 (4 December 2008); De Vries, Karin. 2018. Right to Respect for Private and Family Life. In *Theory and Practice of the European Convention on Human Rights*, ed. Pieter van Dijk et al., 673. Cambridge: Intersentia.

15 ECtHR (GC) Magyar Helsinki Bizottság v. Hungary, appl.no. 18030/11, § 191 (8 November 2016); ECtHR (GC) S. & Marper v. UK, appl.nos. 30,562/04, 30,566/04, § 66–67 (4 December 2008).

16 ECtHR (GC) S. & Marper v. UK, appl.nos. 30,562/04, 30,566/04, § 72, 75, 84 (4 December 2008); ECtHR Aycaguer v. France, appl.no. 8806/12, § 33 (22 June 2017).

17 ECtHR (GC) S. & Marper v. UK, appl.nos. 30,562/04, 30,566/04, § 86, 120 (4 December 2008).

18
*Idem*. at § 72, 120.

19
*Idem.* at § 74–76.

20
*Idem.* at § 71.

21
*Idem.* at § 78, 81, 84, 86.

22 Cf. ECtHR (GC) S. & Marper v. UK, appl.nos. 30,562/04, 30,566/ 04, § 72–73 (4 December 2008).

23 E.g. ECtHR Peruzzo and Martens v. Germany, appl.nos. 7841/08, 57,900/12, § 44–49 (4 June 2013); ECtHR W. v. The Netherlands, appl.no. 20689/08, 9 (20 January 2009); ECtHR Schmidt v. Germany, appl.no. 2352/02 (5 January 2006).

24 ECtHR (GC) Ibrahim and others/UK, appl.nos. 50,541/08, 50,571/ 08, 50,573/08, 40,351/09, § 266 (13 September 2016)..

25
*Idem.*

26
*Idem.*

27 ECtHR (GC) Ibrahim and others/UK, appl.nos. 50,541/08, 50,571/ 08, 50,573/08, 40,351/09, § 267 (13 September 2016).

28 ECtHR GC) Saunders v. UK, appl.no. 19187/91, § 69 (17 December 1996). See more recently: ECtHR Caruana v. Malta, appl.no. 41079/16, § 36 (15 May 2018).

29 ECtHR Funke v. France, appl.no. 10828/84 (25 February 1993); ECtHR J.B. v. Switzerland, appl.no. 31827/96 (3 May 2001).

30 Cf. [47, 48].

31 This could only be different under certain exceptional circumstances, i.e. if excessive forms of physical or psychological pressure are used, often in the form of treatment which constitutes torture, inhuman or degrading treatment in the means of article 3 ECHR [10, 49].

32 ECtHR Funke v. France, appl.no. 10828/84, § 44 (25 February 1993). See also [44, p. 250–251].

33 Cf. ECtHR Wanner v. Germany, appl.no. 26892/12 (23 October 2018).

34 ECtHR (GC) Tommaso v. Italy, appl.no. 43395/09, § 143 (23 February 2017).

35 ECtHR (GC) Boulois v. Luxembourg, appl.no. 37575/04, § 85 (3 April 2012); ECtHR Ganci v. Italy, appl.no. 41576/98, § 20–22 (30 October 2003).

36 In philosophy of mind, even those who hold that mental states are fully determined by brain states typically allow that the two are in some way distinct.

37 ECtHR M. and M. v. Croatia, appl.no. 10161/13, § 171 (September 3, 2015), which had been at stake in cases regarding abortion, euthanasia and recognition of transsexuals.

38 ECtHR (GC) Barbulescu v. Romania, appl.no. 61496/08, § 70.

39 See for an ethical perspective [51].
